# Extraction of Heat-Stabilised Defatted Rice Bran Protein by Solid-State Fermentation Using Heterofermentative Microbes from Traditional Asian Starters

**DOI:** 10.17113/ftb.61.04.23.8255

**Published:** 2023-12

**Authors:** Ugyen Ugyen, Raintong Singanusong, Mahattanee Phinyo, Phanupong Changtor, Sirilux Chaijamrus, Tipawan Thongsook

**Affiliations:** 1 Department of Agro-Industry, Faculty of Agriculture, Natural Resources and Environment, Naresuan University, 65000 Phitsanulok, Thailand; 2 Department of Agricultural Science, Faculty of Agriculture, Natural Resources and Environment, Naresuan University, 65000 Phitsanulok, Thailand; 3 Department of Biology, Faculty of Science, Naresuan University, 65000 Phitsanulok, Thailand

**Keywords:** heat-stabilised defatted rice bran, koji, loog-pang, microbiome diversity, protein extraction, solid-state fermentation

## Abstract

**Research background:**

Heat-stabilised defatted rice bran (HSDRB) is a primary by-product of rice bran oil extraction industry and a nutritious source of protein. However, despite the unique nutritional profile of rice bran protein, the protein-rich by-product, HSDRB is underutilised as a low-value animal feed. Research on protein extraction from HSDRB by enzymatic hydrolysis has attracted the attention of numerous scientists. However, a cost-effective extraction method is required to mitigate the high costs associated with the use of enzymes. Therefore, we have presented an alternative economical and natural approach for protein extraction from HSDRB by solid-state fermentation (SSF) with heterofermentative microbes.

**Experimental approach:**

SSF of HSDRB with two types of traditional Asian fermentation starters, namely loog-pang and koji, were evaluated for enzyme production and their efficacy in extracting proteins from HSDRB. For this purpose, HSDRB fermentation was carried out for 0, 12, 24, 48, 72 and 96 h followed by 24-hour hydrolysis to evaluate the extracted rice bran protein. In addition, microbiome diversity in the fermentation starters was also determined by metagenomic sequencing of 16S rRNA and internal transcribed spacer to identify bacteria and fungi, respectively.

**Results and conclusions:**

The microbial community in the fermentation starters showed the dominance of lactic acid bacteria (LAB) such as *Bacillus subtilis* in loog-pang and *Streptococcus lutetiensis*, *Bacillus pumilus, Lactococcus cremoris, Lactococcus garvieae* and *Pediococcus pentosaceus* in koji, while yeast species *Saccharomycopsis fibuligera* and *Saccharomyces cerevisiae* dominated the fungal diversity in loog-pang and koji starters, respectively. The results suggest that loog-pang and koji can produce cellulase, neutral and acid proteases during fermentation. Despite the discrepancy in their microbial diversity and the enzyme activity during SSF, both starters could effectively increase protein extraction from HSDRB. A positive relationship between the SSF duration and extracted protein was observed. During SSF with loog-pang and koji after 72 h followed by 24-hour hydrolysis, 65.66 and 66.67 % protein was extracted from HSDRB, respectively. The amino acid analysis of the protein hydrolysate produced by the non-fermented and fermented methods showed no difference and had an abundance of glutamic and aspartic acids, leucine, arginine, alanine and glycine amino acids, which accounted for approx. 58 % of the total amino acids.

**Novelty and scientific contribution:**

Loog-pang and koji (traditional Thai and Japanese fermentation starters, respectively) were found to be effective in extracting proteins from HSDRB by SSF although they are inexpensive microbial enzyme sources. Future research aimed at scaling up HSDRB protein extraction for usage in industrial applications can draw on our results.

## INTRODUCTION

Rice bran is an important by-product of rice milling and contains approx. 12-15 % proteins (*1*). It is normally used in the oil extraction which produces heat-stabilised defatted rice bran (HSDRB) as the primary by-product, increasing the protein content in HSDRB to roughly 15–18 % (*2*).

Rice bran protein has an amino acid composition close to the recommendations of the Food and Agricultural Organization (FAO) and the World Health Organization (WHO) (*3*, *4*). The protein efficiency ratio of rice bran (2.37) is reported to be comparable to that of animal casein protein (2.5) with its digestibility over 90 % (*5*). In addition, its hypoallergenic property makes it an ideal ingredient for infant formula with anticancer activity (*6*). However, HSDRB is used as a low-cost ingredient for animal feed and fertilisers. Therefore, HSDRB is expected to change from an undervalued waste to a viable protein source.

Several scientists have reported difficulties in protein extraction from HSDRB due to the protein denaturation caused solely by the heat stabilisation step, as well as the tight bonds that the proteins form with other rice bran components such as phytic acid, carbohydrates and fibre. On the other hand, a plethora of literature has been published on protein production from HSDRB hydrolysis using enzymes such as protease and cellulase. According to Amagliani *et al.* (*7*) and Fabian and Ju (*8*), when enzymes such as cellulase, hemicellulase and xylanase are used, the cell wall components of the bran are broken down and proteins are released from the bonds. The ability of protease to hydrolyse protein into more soluble peptides is used to improve protein recovery and produce a variety of protein hydrolysates with a very high percentage of extracted proteins. Nevertheless, research on HSDRB protein continues. Industrial manufacturers are currently working to reduce the cost of the protein extraction process, in order to minimise the enzyme costs and improve the protein extraction yield. Therefore, the aim of this study is to investigate an alternative method to improve protein extraction from HSDRB by fermentation with heterofermentative microbes.

Solid-state fermentation (SSF) is a well-known green technique for valorising agro-food waste and bioconverting it into value-added products. Therefore, it is often preferred over submerged fermentation and enzymatic hydrolysis as it offers numerous advantages, such as higher economic viability, higher end-product concentration, lower sensitivity to bacterial contamination and the ability to cultivate water-insoluble substrates (*9*). Notably, enzymes, such as cellulase (*10*) and protease (*11*), have also been produced from filamentous fungi with rice bran by the SSF technique, which will help increase and extract the protein in HSDRB. Numerous authors have applied this economical approach in the enrichment and preparation of proteins from various agro-food wastes. According to Kupski *et al.* (*12*), in their work, 26.6 % protein was recovered from rice bran after 120 h of fermentation with *Rhizopus oryzae* CCT 7560, which was about a 49 % increase compared to untreated bran. Recently, Bisly *et al.* (*13*) extracted 64.6 % of total protein by fermenting HSDRB with *Bacillus subtilis* (natto) Takahashi for 61 h. These findings of earlier studies indicate the potential and viability of the SSF in preparing protein extract from different by-products, and the same technique can be applied to prepare HSDRB protein. The filamentous fungi and yeasts commonly used in the fermentation process are usually regarded as safe; *i.e.* their use in SSF results in the production of final products that are free of toxins and thus safe for human and animal consumption (*14*).

SSF with heterofermentative microbes is an alternative approach for protein extraction from HSDRB. Rice cake starter is a traditional starter for alcoholic and fermented foods used in many Asian countries. The starters consist of microorganisms such as yeasts, moulds and bacteria capable of producing hydrolysis enzymes that facilitate the release of proteins by loosening HSDRB structure. In Thailand, rice cake starter, commonly known as ’loog-pang’, is made from alcoholic fermented yeast, starch hydrolysing fungi, herbs, spices and glutinous rice. It has been successfully used to produce alcoholic beverages (*15*, *16*). In other Asian countries, it is known by different local names such as ’Chu’ or ’Chinese yeast cake’ in China and ’koji’ in Japan. Koji is prepared similarly to loog-pang by inoculating koji moulds such as *Aspergillus* sp. or *Rhizopus* sp. on steamed rice or wheat. Koji is capable of producing important hydrolytic enzymes, including amylases, proteases and lipases (*17*, *18*).

The microbial population dynamics in loog-pang differs depending on a production process. Most moulds such as *Aspergillus* sp., *Mucor* sp., *Rhizopus* sp. and *Amylomyces* sp*.,* yeasts such as *Saccharomyces* sp. and *Saccharomycopsis* sp*.,* and some acetic or lactic acid bacteria were identified in the starter (*19*). Due to their low cost and availability, these traditional fermentation starters can serve as effective and inexpensive enzyme sources for protein extraction from HSDRB.

Therefore, in this study, the feasibility of SSF by heterofermentative microbes from loog-pang and koji as the sole enzyme sources was investigated, followed by testing of their effectiveness in protein extraction from defatted rice bran by SSF. We also investigated the microbial diversity in loog-pang and koji starters using a culture-independent approach.

## MATERIALS AND METHODS

### HSDRB and fermentation starter

HSDRB containing on dry mass basis (in %): protein 16.61, moisture 7.8, carbohydrates 52.35, ash 11.51, crude fibre 10.32 and lipid 1.32 was supplied by Surin Bran Oil Co., Ltd. (Surin, Thailand). The bran was packed in polyethylene bags and kept at -20 °C until use in the fermentation process. Before the experiment, HSDRB was sieved through a 20-mesh sieve to obtain uniform particle size. Loog-pang (dried solid of mixed microbial culture) was purchased from local producers in Nan province, Thailand. It contained a total microbial count of 4.60·10^8^ CFU/g and culturable yeast and mould of 1.65·10^8^ CFU/g (method according to Bacteriological Analytical Manual (BAM)). Koji (Koji-kin, Vision Brewing, Nedlands, WA, Australia), a white powder used as sake and miso starter, was purchased from a supplier in Thailand. It contained a total microbial count of 1.75·10^8^ CFU/g and culturable yeast and mould of 1.35·10^8^ CFU/g (BAM method). Koji was stored in a refrigerator at 4 °C, while loog-pang was kept dry at ambient temperature and used within 6 months. All the chemicals used in the experiment for the determination of the amino acid composition were of analytical grade and HPLC grade.

### Isolation and identification of microbiome diversity in fermentation starter

#### DNA extraction, sequencing and analysis

For metagenomic DNA analysis, loog-pang and koji starters were extracted and purified following the instructions of PureLink^TM^ microbiome DNA purification kit (Thermo Fisher Scientific, Waltham, MA, USA) and the samples were stored at -20 °C until use. The purified DNA concentration was determined using a Qubit fluorometer (Thermo Fisher Scientific) and gel electrophoresis was used to determine the presence of genomic DNA. The metagenomic sequencing of 16S or internal transcribed spacer (ITS) preparation followed similar standard guidelines of the 16S metagenomic sequencing library preparation protocol of the Illumina MiSeq System (Illumina, San Diego, CA, USA), but with different primers. The V3-V4 segment of bacterial 16S rRNA was amplified with forward (5’-CCTACGGGNGGCWGCAG-3’) and reverse (5’GACTACHVGGGTATCTAATCC-3’) primers. For fungi, the ITS segment was amplified with forward (5′-CTTGGTCATTTAGAGGAAGTAA-3′) and reverse (5′-GCTGCGTTCTTCATCGATGC-3′) primers. Sequencing was performed on a MiSeq system (Illumina). For bacterial and fungal data preparation, raw sequence reads of approx. 300 bp were analysed with MiSeq reporter and the Greengenes database (*20*) was used to classify the genus and species of the microbiome diversity of the sample.

#### Species identification of the microbes in the fermentation starter and its visualisation

The FASTQ data were then converted into the FASTA format and fasqutils was used to analyse the quality score. The raw whole metagenome shotgun sequencing reads of each sample dataset were trimmed using SeqPrep (*21*) and Sickle (*22*) based on the sequence length and quality. Using default parameters, MetaPhlAn 3 (*23*) was used to calculate the taxonomic profile of the microbiome at the species level for each sample. The search engine used was Bowtie2 v. 2.2.9 (*24*). The percentage identities were determined using the BLASTn tool (https://blast.ncbi.nlm.nih.gov/Blast.cgi), which is based on the 16S data sequence for bacteria and the ITS data sequence for fungi. The R packages *ape* (*25*) and *metacoder* (*26*) were used to make the heat tree and the meta diversity plots that show the results. The heat tree function generates a tree where sequence abundance statistics are shown as colours and sizes for each taxon. Each taxonomic hierarchy and abundance is shown as a different colour and size of nodes.

### Solid-state fermentation of HSDRB

SSF was carried out in a 200-mL glass jar with 10 g HSDRB moistened with 10 mL deionised water and sterilised at 121 °C for 20 min using an autoclave (HV-5011; HIRAYAMA, Saitama, Japan). The autoclaved mixture was cooled overnight to room temperature and inoculated with (on a dry mass basis) *w*(loog-pang powder)=1.7 % or *w*(koji culture)=0.4 % according to the recommendations of the producers. The inoculated solution was then thoroughly mixed with alcohol- and heat-sterilized glass rod and cultured at 30 °C for 0, 12, 24, 48, 72 and 96 h. All the fermentations were conducted in duplicate including the naturally fermented sample prepared in the same manner without the addition of a starter. The pH of the samples was measured using a digital pH meter (starter 3100; Ohaus, Parsippany, NJ, USA) and the reducing sugar content was determined according to Miller (*27*).

### The extraction and evaluation of enzymes

The fermented HSDRB obtained after different fermentation time (0, 12, 24, 48, 72 and 96 h) was added to 50 mL of NaCl (0.9 %) solution and the enzyme extract was prepared under orbital shaking (28L-M; PolyScience, Niles, IL, USA) at 200×*g* and ambient temperature (37 °C) for 60 min. The supernatant obtained after filtration and centrifugation (Z326K; Hermle, Gosheim, Germany) at 3000×*g* for 30 min was used as the enzyme extract. One unit of enzyme activity was defined as the amount of cellulase and protease enzyme required to produce colour equivalent to 1 µmol of reducing sugar and tyrosine in 1 min, respectively, under the assay conditions.

### Cellulase activity of defatted rice bran

The cellulase activity was analysed according to Denardi de Souza *et al.* (*28*) using carboxymethyl cellulose (0.5 %) substrate in 0.05 M sodium acetate buffer (pH=4.8) at 50 °C for 30 min. The reducing sugar quantity was determined by the 3,5-dinitrosalicylic (DNS) acid using a glucose (1 mg/mL) standard curve.

### Neutral and acid protease activity of defatted rice bran

The neutral and acid protease activities were evaluated using 2 % (*m*/*V*) casein prepared in 0.2 M sodium phosphate (pH=7) and 2 % (*m*/*V*) bovine haemoglobin prepared in 0.5 M sodium acetate (pH=4.8) as the substrate for neutral and acid protease, respectively.

The reaction of the mixture of substrates and enzyme extract was carried out at 37 °C for 30 min according to Su *et al.* (*29*). An l-tyrosine solution (0.2 mg/mL) was used to prepare a linear standard curve.

### Preparation of HSDRB protein hydrolysate

The HSDRB fermented for different times was added to deionised water in the ratio 1:6 (*m*/*V*) and hydrolysed for 24 h at 200×*g* and 55 °C using an orbital shaker (28L-M; PolyScience). Then, it was filtered using a nylon filter bag (0.2 µm) to remove the remaining fungal cells and spores along with the fermented biomass. The obtained filtrate was pooled together with the liquid obtained after washing the residue with boiled deionised water in the ratio 1:6 (*m*/*V*). The mixture was boiled for 10 min to inactivate the microbial enzymes and then dried at 60 °C to obtain the solid protein. The dried HSDRB protein hydrolysate was then ground into a fine homogeneous powder of 0.6 mm mesh screen, stored in a sealed container and kept inside the desiccator for further analysis.

### Determination of the extracted protein

The protein mass fraction in the hydrolysates was determined by measuring the total amino nitrogen according to the Kjeldahl method using 5.95 as a conversion factor (*13*). The extracted protein was expressed in % on dry mass basis as the ratio of the mass of protein (g) in the sample to the initial mass of protein (g) in the HSDRB used for the SSF.

### Sodium dodecyl sulfate-polyacrylamide gel electrophoresis (SDS–PAGE)

SDS-PAGE of HSDRB protein and its hydrolysate was performed according to a method previously described by Laemmli (*30*) using a 15 % polyacrylamide separating gel. Protein samples (0.05 g) dissolved in deionised water (100 µL) were mixed with 4× loading buffer (3:1, *V*/*V*) containing 0.1 M Tris-HCl, pH=6.8, 2 % SDS, 5 % β-mercaptoethanol and 15 % glycerol and then heated for 5 min in boiling water. Samples (15 µL) were loaded and electrophoresis was carried out in a Mini-Protean Tetra Cell (Bio-Rad, Hercules, CA, USA) at a 100 V constant current for approx. 180 min. After electrophoresis, the gel was stained with Coomassie brilliant blue R-250 and destained with ethanol and acetic acid. The protein molecular mass was determined using a protein marker with molecular mass ranging from 10 to 250 KDa (Bio-Rad).

### Determination of amino acid composition

Non-fermented and fermented HSDRB protein and its protein hydrolysate were hydrolysed with 6 M HCl at 110 °C for 22 h. Amino acids were determined with high-performance liquid chromatography (HPLC) (Waters Alliance 2965; Milford, MA, USA) with the following HPLC settings: at 35 °C, using the Poroshell C18 column (4.63 mm×100 mm×2.7 µm; Agilent Technologies, Santa Clara, CA, USA), a flow rate of 1.2 mL/min, gradient program, mobile phase acetonitrile, sodium acetate buffer and water, and fluorescence detector (Jasco FP2020; Hachioji, Tokyo, Japan) at *λ*=395 nm.

### Statistical analysis

All determinations were done in duplicate and the obtained data were analysed using a one-way ANOVA. Duncan’s new multiple range tests were applied to determine the significant difference between samples at a 95 % confidence level using SPSS v. 23 software (IBM, Armonk, NY, USA).

## RESULTS AND DISCUSSION

### *Microbiome diversity in the fermentation starter*s

The microbial communities in loog-pang and koji were identified by metagenomic sequencing of 16S rRNA and ITS segments. The metagenomic DNA sequencing approach is a culture-independent high-throughput method and can identify microbial communities including both culturable and non-culturable microbiomes with high accuracy within a short time (*31*). Considering both fermentation-relevant microbes and other microbial taxa, a total of 90 species including both bacteria and fungi were identified in loog-pang and koji starter samples. Loog-pang contained a total of 48 bacterial and 3 fungal species, while koji contained 34 bacterial and 5 fungal species.

**Fig. 1:**
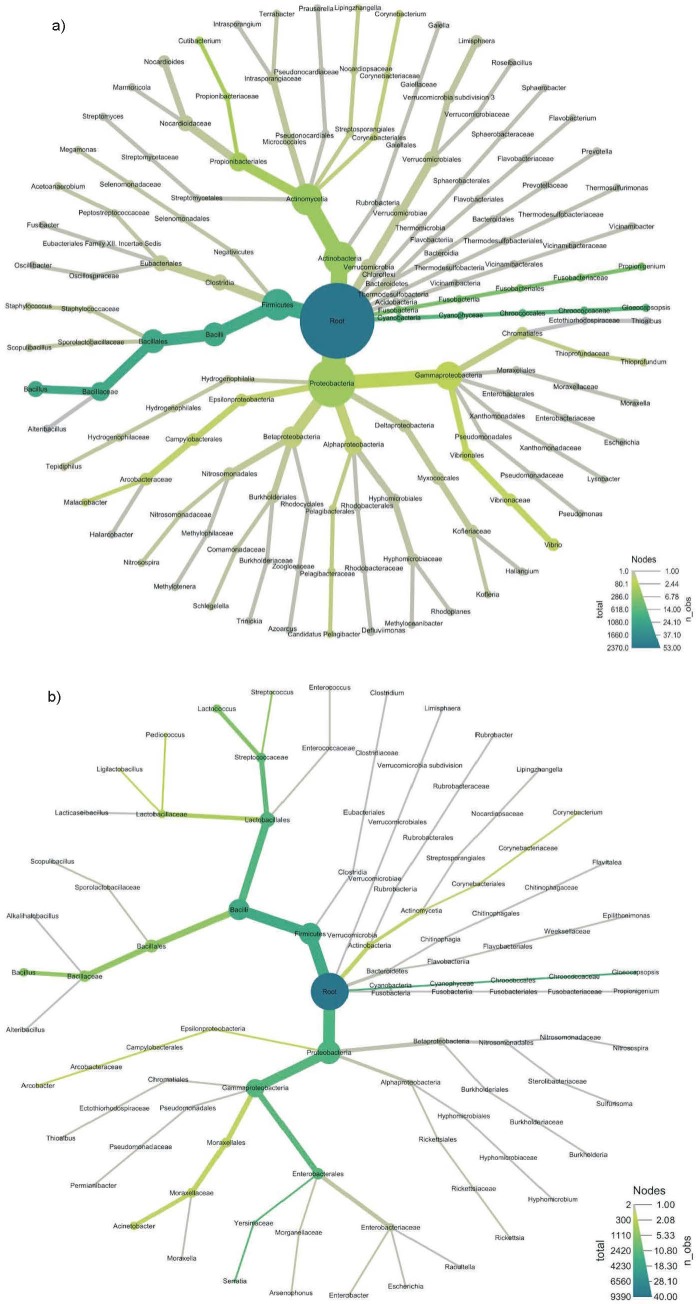
Heat tree showing taxonomic comparisons of: a) loog-pang and b) koji. The metagenomic plot summarises the taxonomic assignments obtained for all sequences of bacterial 16S rRNA, where node size correlates with the abundance

**Fig. 2:**
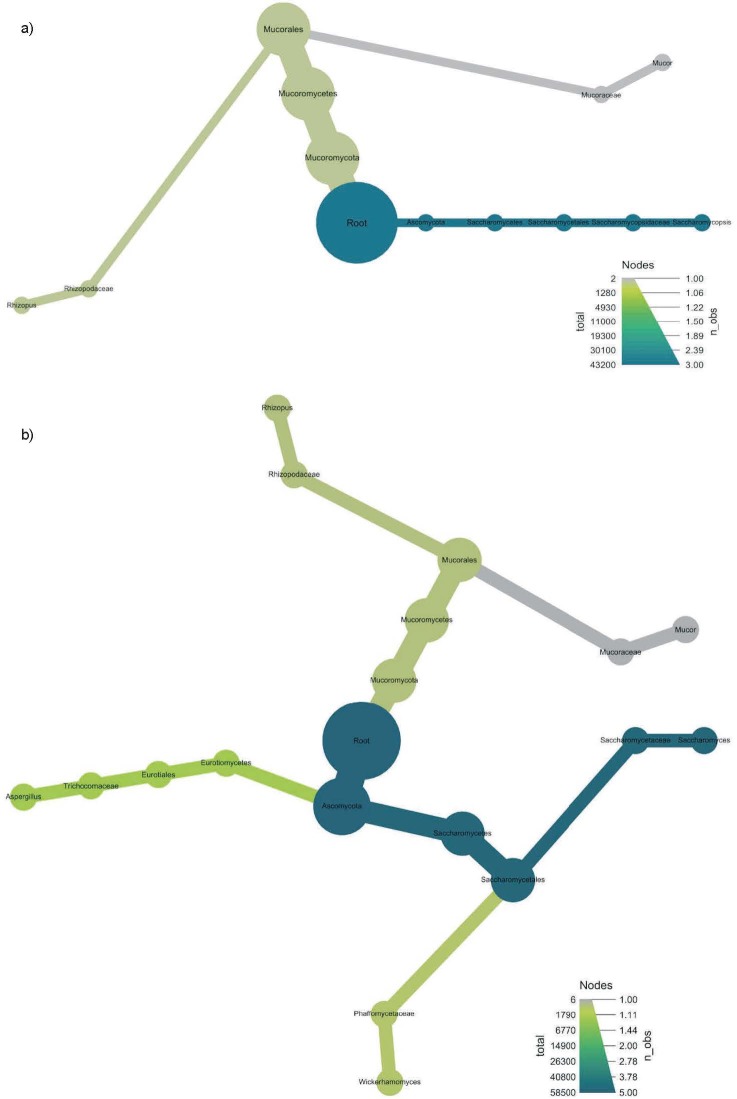
Heat tree showing taxonomic comparisons of: a) loog-pang and b) koji. The metagenomic plot summarises the taxonomic assignments obtained for all sequences of ITS of fungi, where node size correlates with the abundance

The heat tree shown in Fig. 1 and Fig. 2 illustrates the taxonomic hierarchy of the microbial community in loog-pang and koji starters. The nodes of the tree represent the taxonomic levels, and each branch illustrates the link between the various entities. The abundance of operational taxonomic units (OTU) is expressed by the size, colour and colour intensity of the nodes. Nodes that are darker green have a higher abundance, while nodes that are lighter green have a lower abundance of microorganisms. Therefore, koji was substantially enriched with *Cyanobacteria, Proteobacteria* and *Firmicutes.* The estimated mean OTU abundance of the persistent phylum in the top of the tree was significant (p<0.001). On the other hand, the abundance of *Fusobacteria* in loog-pang was similar to the relative abundance of persistent phylum in both samples in the top of the tree.

The fungal community in both starters had a significantly higher (p<0.001) estimated mean relative abundance of phylum *Ascomycota* and the estimated mean OTU abundance of phylum *Mucoromycota* was significantly lower than the phylum *Ascomycota* (p<0.001) in both koji and loog-pang samples.

**Fig. 3:**
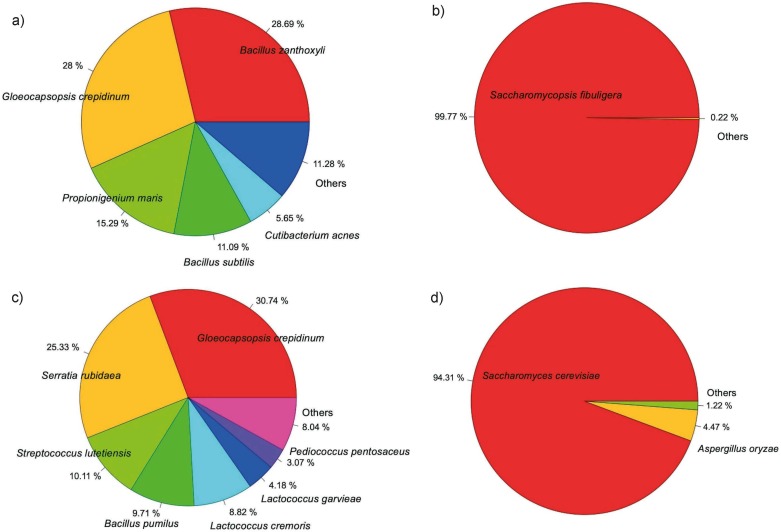
Abundance of microbial diversity in: a) bacteria and b) fungi in loog-pang, and c) bacteria and d) fungi in koji starter

Fig. 3 shows the abundance of lactic acid bacteria (LAB) such as *Bacillus subtilis* in loog-pang and *Streptococcus lutetiensis*, *Lactococcus cremoris, Lactococcus garvieae*, *Pediococcus pentosaceus* and *Bacillus pumilus* in koji, while yeast species *Saccharomycopsis fibuligera* and *Saccharomyces cerevisiae* dominated the fungal diversity and showed their high abundance in loog-pang and oji starters, respectively. *Aspergillus oryzae* was also identified in the fungal diversity of koji.

### pH and reducing sugar mass fraction during SSF

**Fig. 4:**
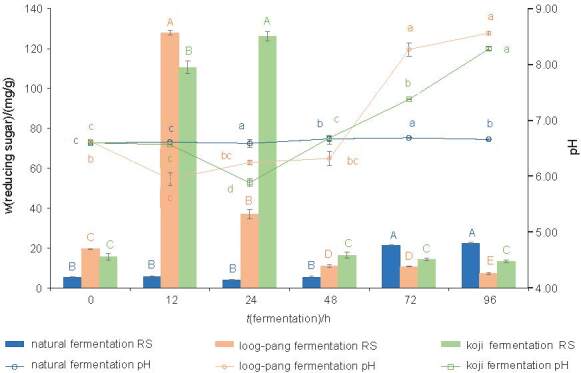
The pH values and reducing sugar mass fraction on dry mass basis as a function of the time of solid-state fermentation (SSF). Different lower-case letters for pH change and upper-case letters for reducing sugar in the graph indicate a significant difference (p≤0.05) as a function of fermentation time using Duncan’s test

The change in the pH and reducing sugar mass fraction during the SSF of HSDRB in response to the metabolic activities of the microorganisms from loog-pang and koji is shown in Fig. 4. A contradictory relationship between the pH and the reducing sugar mass fraction was observed as the maximum reducing sugar mass fractions of 127.9 and 126.3 mg/g of defatted rice bran were produced in fermentations with loog-pang and koji at the lowest pH of 5.95 and 5.89 after 12 and 24 h, respectively. The pH decreased due to the microorganisms digesting the nutrients for their growth, producing alcohol and organic acids such as lactic acid and other metabolites. This led to the degradation of HSDRB polysaccharides, most likely starch cellulose and hemicellulose, into soluble reducing sugars. The overall trend of reducing sugar content, *i.e.* initial increase followed by a gradual decrease, during the SSF was similar to the results reported by other authors (*32*, *33*).

### Cellulase activity

**Fig. 5:**
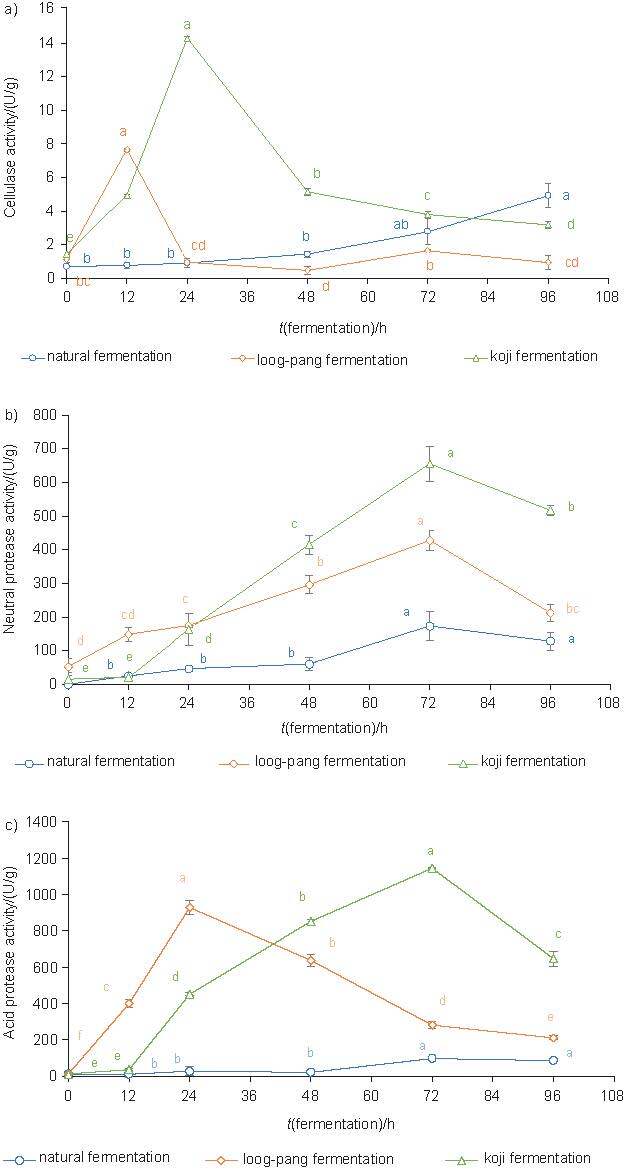
The results of the activities of: a) cellulase, b) neutral protease and c) acid protease as a function of solid-state fermentation time. Different letters indicate significant differences (p≤0.05) as a function of fermentation time using Duncan’s test

Cellulase breaks down β-1,4-glycosidic bonds of cellulose and produces reducing sugar/glucose monomers. *Aspergillus* sp. (*34*), *Saccharomyces cerevisiae* and *Saccharomycopsis fibuligera* (*35*) have been reported to produce cellulase. Considering the identification of these species in this study, cellulase production was monitored with HSDRB under SSF and shown in Fig. 5a. It was found that loog-pang and koji produced cellulase enzymes to degrade the cellulose polysaccharides from the HSDRB substrates to sugars for easy assimilation and growth support.

In general, enzyme activity increases as fermentation progresses, but decreases as nutrients are consumed (*36*). A similar effect was also obtained in this study, with activity initially increasing and then decreasing with increasing SSF time. The maximum cellulase activity was reached after 12 h at 7.65 U/g and after 24 h at 14.29 U/g in fermentation of loog-pang and koji, respectively. Accordingly, the peak reducing sugar production was also measured at this time (Fig. 4). This observation that the maximum activity is responsible for the highest amount of reducing sugar produced was similar to Khan *et al.* (*37*). In addition to glucose, xylose, arabinose and xylooligosaccharides contributed to the increase in reducing sugars as they were products of the activity of the enzyme on cellulose and arabinoxylan, the most common hemicelluloses in rice bran. However, the significant decrease in the activity after 12 or 24 h in loog-pang and koji, respectively, was partly due to the depletion of cellulose and hemicelluloses. The changes in the culture environment indicated by the increase in pH could not favour the growth of the bacteria, resulting in the decrease in the activity.

### Neutral and acid protease activity

Filamentous fungi and yeasts such as *Aspergillus oryzae* (*38*), *Saccharomycopsis fibuligera* (*39*) and *Saccharomyces cerevisiae* (*40*), which were identified in this study, can also produce proteases. The optimal activity of these proteases depends on their pH range, which is 6.0-8.0 for neutral proteases and 2.0-6.0 for acid proteases. However, the neutral protease has a dominant hydrolysis efficiency over all other proteases (*41*). Therefore, the activity of neutral and acid proteases during SSF was monitored in this study, as shown in Fig. 5b and Fig. 5c, respectively.

The neutral protease activity pattern showed that the activity increased with time and, interestingly, the changing patterns of neutral protease indicated the different growth phases of the collective culture strains in loog-pang and koji: 0–12 h for spore germination, 12–72 h hyphal growth and 72–96 h for sporulation (*41*, *42*). However, in natural fermentation, the initial phase of spore germination was longer (0–48 h). Maximum activity was observed in all cultures after 72 h, with koji (655.52 U/g) having higher activity than loog-pang (427.57 U/g). At the beginning of fermentation, the activity was low as the fungal spores of loog-pang and koji adapted to the culture environment, started to germinate and prepared for enzyme production. As the growth of hyphae started (after 12 h), the release of degradation enzymes also started, which converts large molecules like proteins and carbohydrates into small absorbable molecules to support growth and energy. After a while, the activity decreased, probably due to moisture loss, substrate deficiency, suppression of catabolism, pH variations and production of amino acids and low-molecular-mass compounds (*43*, *44*). A similar trend was also observed by Sumantha *et al.* (*45*) with a maximum protease activity of 129 U/g dry solid substrates after 72 h of fermentation of *Rhizopus microsporus* NRRL 3671 on rice bran substrates.

Acid proteases were also detected during SSF of HSDRB (Fig. 5c). The maximum activity was observed with loog-pang after 24 h and with koji after 72 h during natural fermentation. However, the pH during fermentation was in the neutral pH range, as reported in Fig. 4. This indicated that acid protease was probably not involved in the protein hydrolysis as the fermentation pH was not in its optimal range. Therefore, the present result indicates the dominance of neutral protease activity for the growth and metabolism of the loog-pang and koji culture.

### Extracted HSDRB protein hydrolysate

The extracted protein from HSDRB hydrolysate shows that both loog-pang and koji improved protein extraction, as shown in Table 1. The extraction of protein significantly (p≤0.05) increased with fermentation time, while natural fermentation resulted in much less extracted protein (data not shown) because of low microbial growth and less produced enzymes (Fig. 5). Both cultures could extract maximum protein from HSDRB after 72 h, namely 65.66 and 65.67 % with loog-pang and koji starter, respectively. The effect of both starters on protein extraction from HSDRB was comparable to that of *B. subtilis* (natto) Takahashi reported by Bisly *et al.* (*13*). The extracted protein of 64.6 % was obtained from HSDRB fermented with *B. subtilis* (natto) Takahashi culture for 61 h in SSF.

**Table 1:** Mass fractions of extracted proteins during fermentations of loog-pang and koji

*t*(fermentation)/h	*w*/(extracted protein)/%	
Loog-pang fermentation	Koji fermentation
0	(35.8±3.0)^d^	(29.9±2.5)^c^
12	(47.6±0.2)^c^	(31.9±0.3)^c^
24	(43.2±0.5)^c^	(61.3±0.6)^b^
48	(57.6±2.6)^b^	(61.4±0.6)^b^
72	(65.7±1.2)^a^	(65.7±2.2)^ab^
96	(66.7±1.7)^a^	(68.9±4.3)^a^

Results are presented as mean value±standard deviation, *N*=2. Different letters in superscript in the same column indicate significant differences (p≤0.05) between the samples using Duncan’s test

The production of protein hydrolysate with a large amount of protein from defatted rice bran or HSDRB has been reported by several authors (*46*-*49*) using enzymatic hydrolysis with proteases, cellulase, Alcalase and phytase. The use of these enzymes facilitates intercellular protein release from the cell matrix of defatted rice bran and proteolytic hydrolysis efficiently hydrolysed the proteins released into peptides, improving their solubility, which can be used for their extraction. Similarly, the cellulase produced at different stages of microbial metabolism in this study hydrolysed and consumed the cellulosic polysaccharide matrix in defatted rice bran, releasing the proteins bound in this matrix. Consequently, the proteolytic hydrolysis further increased the protein content in the DRBPH. It was found that the extraction of protein increased with increasing hydrolysis time. The protein extraction of 39.23, 43.10 and 55.57 % was obtained after SSF with different hydrolysis times of 6, 12 and 24 h, respectively.

**Fig. 6:**
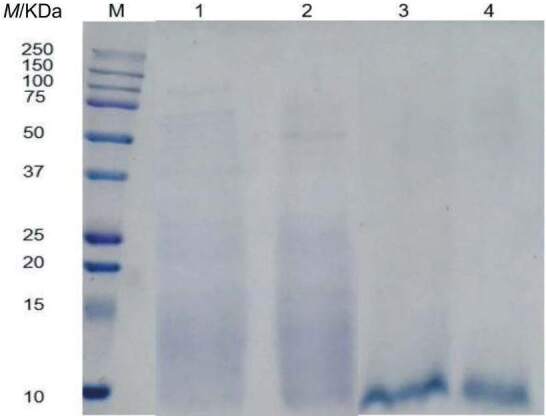
SDS-PAGE profile of defatted rice bran protein. M=marker, 1 and 2=koji and loog-pang defatted rice bran protein, respectively, 3 and 4=koji and loog-pang defatted rice bran protein hydrolysate, respectively. All samples (8 %, *m/V*) were mixed with the sample buffer

Fig. 6 shows the SDS-PAGE profile of HSDRB protein. The fermented defatted rice bran proteins in both loog-pang and koji consisted of polypeptides of both small to medium and large size protein bands ranging from 10 to 100 kDa. However, after hydrolysis, all the large and medium-sized proteins in fermented defatted rice bran were fragmented into lower molecular mass peptides. The obtained result suggests that hydrolysis could effectively break down larger protein fragments of fermented defatted rice bran proteins into smaller molecular mass peptides and hence improve their solubility and enhance the protein content in the defatted rice bran protein hydrolysate.

Although loog-pang and koji produce cellulase and protease enzymes with different activities, they showed similar effects on the extracted protein (~66 %). This is likely because the carbohydrase responsible for releasing the protein from the defatted rice bran matrix did not cause a significant difference in protein extraction, in which is consistent with other studies. Tang (*48*) and Jodayree *et al.* (*50*) indicated that the addition of Celluclast at different concentrations had no significant effect on protein extraction from bran waste such as HSDRB and defatted oat bran. However, amylase in combination with protease has been reported to extract about 61 % protein from HSDRB (*51*), and accordingly, the combined effect of cellulase and protease produced in this study also suggests the same. Moreover, the release of fermentation metabolites, especially similar reducing sugar mass fractions (Fig. 4) during SSF suggests a similar hydrolysis rate of cellulase produced by each starter. Although SSF with loog-pang induced fewer proteases than koji, it seemed that the proteases produced during the fermentation by loog-pang were sufficient for proteolytic activity. As a result, an extracted protein of ~66 % was obtained in the hydrolysates in the fermentation of both loog-pang and koji. The extracted protein yield was much higher than that of Tang (*47*), who extracted 45.4 % protein from HSDRB with amylase, which had a proteolytic activity. The probable reason for this could be the combined effect of proteolytic hydrolysis (*52*) along with the cellulase produced by the fungus, which improved the protein degradation and extraction.

Thus, in this study, defatted rice bran protein hydroysate with a high percentage of extracted protein was successfully produced and SSF with mixed microbial starters was shown as a potential approach for the extraction and production of hydrolysates from other agro-food wastes.

### Amino acid composition

**Fig. 7:**
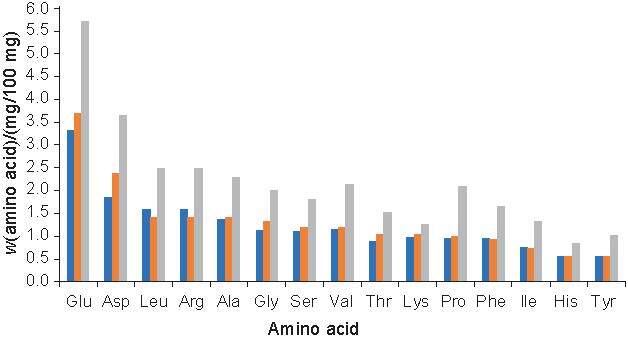
Mass fractions of amino acids in defatted rice bran protein (DRBP). Non-fermented DRBP was solubilised in deionised water followed by precipitation at pH=4. Fermented DRBP was obtained after 72 h of fermentation with loog-pang and hydrolysate of DRBP, DRBPH was obtained after 72 h of fermentation and 24 h of hydrolysis

The amino acid composition of proteins in any food material affects its nutritional and physicochemical properties. Rice bran protein consists of glutamic acid, aspartic acid, leucine and arginine as the most abundant amino acids, accounting for 61 % of the total amino acid content (*53*). The mass fractions of amino acids in non-fermented and fermented defatted rice bran proteins and its hydrolysates are shown in Fig. 7. The results also show the dominance of glutamic acid, aspartic acid, leucine, arginine, alanine and glycine content, which accounted for 57.96 % of the total amino acid mass fraction (18.71 mg/100 mg) in non-fermented defatted rice bran proteins. The high content of flavour enhancers glutamic acid, followed by aspartic acid, which is a key component in the production of artificial sweeteners, and aspartame could have been due to their abundance in the plant protein.

During fermentation, the produced extracellular protease hydrolysed defatted rice bran protein into smaller peptides for microbial use. As a result, the total amino acid mass fraction increased after fermentation and reached 19.85 mg/100 mg. Despite the increase in the amount of extractable proteins, the amino acid composition of the fermented defatted rice bran protein and its hydrolysate remained unchanged, with glutamic acid, aspartic acid, leucine, arginine, alanine and glycine still accounting for 58.55 and 57.81 % of total amino acids, respectively.

However, further hydrolysis of fermented defatted rice bran protein accelerated and fragmented the exposed proteins, thereby improving and increasing the amino acid content in the defatted rice bran protein hydrolysate. The total amino acid mass fraction in the defatted rice bran protein hydrolysate reached 32.26 mg/100 mg, which was an increase of 62.50 and 72.44 % compared to the non-fermented and fermented defatted rice bran protein, respectively. The fact that the fermented defatted rice bran protein contained more amino acids and had the same amino acid composition as the non-fermented defatted rice bran protein showed the potential of SSF in protein extraction, which helped to increase protein production without changing the amino acid composition. Considering the improved amino acid content, the HSDRB protein can also be used to fortify food items such as bread and cookies, as well as improve and enhance the flavour of foods like soup, sauce and poultry.

## CONCLUSIONS

The ability of loog-pang and koji to extract proteins from heat-stabilised defatted rice bran (HSDRB) by solid-state fermentation (SSF) was investigated in this study. The results show that despite different microbial communities, loog-pang and koji can secrete crucial enzymes with almost identical efficacy in improving protein extraction from HSDRB compared to natural or any other extraction methods. Loog-pang and koji were able to extract 65.66 and 65.67 % of proteins with lower molecular mass, respectively, after 72 h of fermentation followed by 24 h of hydrolysisof without altering the composition of amino acids in rice bran protein. The SSF with loog-pang and koji is an effective and inexpensive technique for adding value to HSDRB. This technique enables the upscaling of the protein extraction from HSDRB, which helps to extend the list of applications of HSDRB in the food industry.
